# Social Modulation of Contagious Yawning in Wolves

**DOI:** 10.1371/journal.pone.0105963

**Published:** 2014-08-27

**Authors:** Teresa Romero, Marie Ito, Atsuko Saito, Toshikazu Hasegawa

**Affiliations:** 1 Department of Cognitive and Behavioral Sciences, The University of Tokyo, Tokyo, Japan; 2 Japan Society for the Promotion of Science, Tokyo, Japan; University of Bologna, Italy

## Abstract

On the basis of observational and experimental evidence, several authors have proposed that contagious yawn is linked to our capacity for empathy, thus presenting a powerful tool to explore the root of empathy in animal evolution. The evidence for the occurrence of contagious yawning and its link to empathy, however, is meagre outside primates and only recently domestic dogs have demonstrated this ability when exposed to human yawns. Since dogs are unusually skilful at reading human communicative behaviors, it is unclear whether this phenomenon is deeply rooted in the evolutionary history of mammals or evolved *de novo* in dogs as a result of domestication. Here we show that wolves are capable of yawn contagion, suggesting that such ability is a common ancestral trait shared by other mammalian taxa. Furthermore, the strength of the social bond between the model and the subject positively affected the frequency of contagious yawning, suggesting that in wolves the susceptibility of yawn contagion correlates with the level of emotional proximity. Moreover, female wolves showed a shorter reaction time than males when observing yawns of close associates, suggesting that females are more responsive to their social stimuli. These results are consistent with the claim that the mechanism underlying contagious yawning relates to the capacity for empathy and suggests that basic building blocks of empathy might be present in a wide range of species.

## Introduction

Empathy, the ability to share the feelings and sensations of others, is essential to engage in successful social interactions, coordinated activity, and cooperation toward shared goals [1]. Current evolutionary evidence suggests that empathy is a phenomenon with many intermediate forms, ranging from mere agitation at the distress of others to complex forms of perspective taking [2]–[6]. The data also suggest that empathy might be phylogenetically ancient [1]. However, evidence remains meagre, especially in non-primate species, and more data are needed from a wider range of taxa to better understand the evolution and complexity of empathic abilities in non-human animals. In this respect, contagious yawning, i.e., yawning after seeing or hearing another individual yawn, is an ideal candidate behavior to explore basic forms of empathy across species and different types of social systems.

Although contagious yawning is not in itself an emotional reaction, its occurrence has been clinically, psychologically, neurobiologically, and behaviorally linked to our capacity for empathy. For instance, in humans, contagious yawning has been reported to occur more frequently in individuals who score higher on questionnaires evaluating empathy [7] and less in clinical populations characterized by impaired empathic abilities such as autistic and schizotypic individuals [7], [8]. Yawning when seeing other people yawn has also been associated with activations in the same neural networks responsible for empathy and social skills, such as the ventromedial prefrontal cortex [9]–[11]. Finally, the mirror neuron system [12], [13] is activated when a person views or hears a yawn [10], [11], [14], though the role this system plays in eliciting the actual contagious event remains unclear.

Humans are not the only species that show contagious yawning. Recent studies in non-human primates have further supported the association between contagious yawning and empathy. Chimpanzees (*Pan troglodytes*), bonobos (*Pan paniscus*) and gelada baboons (*Theropithecus gelada*) have been reported to yawn in response to perceiving a conspecific yawning [15]–[20]. In these primate species, as well as in humans [21], yawn contagion occurs more frequently between individuals with a close social bond. These findings fit the empathy-based hypothesis of contagious yawning since similarity, familiarity, and closeness are known to facilitate empathy in both humans and non-humans [1], [4].

The evidence of contagious yawning, as well as its link to empathy, remains limited outside the primate order. Attempts to test the empathy-based, emotionally connected hypothesis of contagious yawning have only been done in the domestic dog *(Canis lupus familiaris)*. Although initial exploration of this phenomenon yielded contradicting results [22]–[24], more recent findings are consistent with the view that dogs are not only able to yawn contagiously - at least when the stimulus presented is a live human yawn [22], [25], [26] - but also that their susceptibility to yawns is affected by the emotional proximity to the yawner. Two independent studies, one using audio stimuli [27] and another using visual stimuli [25], showed that dogs yawned more frequently after being exposed to familiar than to unfamiliar yawns.

That contagious yawning fits predictions derived from the empathy-based hypothesis in two phylogenetically distant species within the Mammalia class could suggest that the link between contagious yawning and empathy is deeply rooted in the evolutionary history of mammals. Alternatively, this could also be the result of convergent evolution. Unlike non-human primates or other canids, domestic dogs are unusually skilled at reading human social and communicative behaviors [28], [29]. For example, dogs show, to some extent, an understanding of human referential intentions expressed in communicative gestures [30], [31], and they respond to what humans can and cannot see in various situations [32]. Thus, it could be possible that dogs' ability to yawn contagiously evolved with the capacity for reading human communicative signals, representing a case of convergent social evolution between primates and dogs. Intriguingly, no study has demonstrated dog-to-dog contagious yawning [23], [24], suggesting that dogs may be predisposed to respond more intensively, or only, to human social cues rather than to those of conspecifics.

We studied the evolutionary emergence of contagious yawning and its link to empathy in mammals by examining the phenomenon in wolves (*Canis lupus lupus*). The wolf is an ideal model species to explore this phenomenon because it is the dog's closest phylogenetic relative and a highly social and cooperative species [33]. If contagious yawning is shared by other social mammals, we would expect it to be present in the wolf. In contrast, the absence of contagious yawning, or its link to empathy, in wolves would suggest that dogs' ability is an evolutionary novel skill, providing a case of behavioral convergence with primates. Using a highly standardized observational approach [19], we specifically investigated under naturalistic settings whether yawning is contagious in wolves and whether this response is biased toward close social partners, as the empathy-based hypothesis predicts.

## Methods

### Ethics statement

The study was conducted in strict accordance with the Guidelines for the treatment of animals in behavioral research and teaching by the Animal Behavior Society/Association for the Study of Animal Behaviour. The study was a purely observational and research permission was obtained from the directors of the Tama Zoological Park belonging to the Tokyo Metropolitan Zoological Park Society. The protocol was approved by the Committee on the Ethics of Animal Experiments of The University of Tokyo (Approval No. 24–23).

### Subjects and Enclosure

Observations were done on a pack of 12 captive wolves (*Canis lupus lupus*) at Tama Zoological Park, Tokyo, Japan. The pack is what would be called a “nuclear family” [33] consisting of the alpha male and female breeding pair and all of their offspring (5 females and 5 males) (Table S1 in File S1). All subjects were adults (i.e. > 2 yr.) at the start of the study and none of the subjects showed any stereotypic or aberrant behavior. The pack was kept in an outdoor enclosure of approximately 250 m^2^ resembling a rocky terrain with cliffs and slopes. Wolves also had access to an inside area of about 50 m^2^. The wolves were fed with meat scattered on the ground once a day and water was available *ad libitum*.

### Data Collection

A total of 254 hours of observation was conducted over a span of 5 months. Following Palagi et al. [19], we recorded any yawns that occurred in absence of external stimuli, such as loud noises or presence of visitors, that could alert the attention of the first yawner or other subjects in the group, and when the observed individuals were awake, in a relaxed situation, either sitting down or roaming, and without visible signs of stress (e.g., self-directed behaviors such as self-scratching or self-touching). When an individual yawned in this context, 1) the exact time of the yawn, 2) the identity of the initial yawner (hereafter the “trigger”), 3) the identity of subjects within two body lengths of the trigger with their eyes open (hereafter the “observer”), and 4) the head orientation of the observer in relation to the trigger (i.e., directly facing the yawner, within sight but not facing the yawner, or completely not seeing the yawner) were recorded. Immediately after the initial trigger's yawn, a three min focal observation on the observer was conducted (i.e. yawn observation), and all yawns performed by the focal subject, along with their latency, recorded. During the yawn observation, we also recorded the total number of yawns performed by the trigger as a measure of the number of opportunities the observer had to observe the stimuli. The whole yawn observation had to occur when the animals were not engaged in feeding, agonistic, sexual, or play behavior.

To match for the original yawning period, a 3 min control period was set on the next possible day, within a 30 min time window of the original yawning time and within a maximum of 10 days. Control observations followed the same sample procedure as yawn observations, with the difference that the observer had to be free of any yawn influence at least 10 min prior to the start of the control observation. Furthermore, if the trigger or any nearby individual other than the focal subject (i.e. the observer) yawned prior to or during the 3 min control period, the observation was cancelled and a new control observation was rescheduled. Spatial proximity and body orientation towards the initial yawner were also matched. If the observer maintained the original situation for at least 10 seconds, the 3 min control period started and any yawns performed by the focal subject were recorded.

Additionally, scan-sampling was conducted every 15 min to collect data on proximity (i.e., body contact; within one body length) and affiliative interactions (i.e., licking, sniffing, playing, head rubbing) for all dyads, along with *ad libitum* data collection on affiliative and agonistic interactions (i.e., whimpering, fleeing, standing over, push, knock-down, growl, gape, charge, chase, wrestle, bite), and submissive/dominant displays (e.g., active submission, passive submission, mouth grasp, Table S2 in File S1) [33].

### Data Analysis

We statistically tested the occurrence of contagious yawning in wolves via a Wilcoxon signed rank test. This analysis compared at individual level the frequency of yawns performed by the observer after the trigger's initial yawn (i.e., yawn observation) with the frequency of yawns performed without the presence of previous yawns (i.e., control observation). Additionally, a generalized linear mixed model analysis (GLMM) with a binomial error structure and a logit link function was conducted to examine the effect of different factors on the occurrence (i.e. presence or absence) of contagious yawning. The dependent variable was a binary term of whether the observer yawned or not, and observer and trigger identities were included as random factors. As fixed factors we included: the observational situation (yawn observation, control observation), the trigger's and subject's sex, head orientation (facing, within sight, not within sight), dominance relationship, spatial proximity (no-close, close), and social bond (weak, strong). The spatial proximity level between dyads was categorized using the proximity data collected during scans and calculating the quartile points of dyadic scores for each focal individual. We considered dyads in the upper quartile as dyads sharing a close proximity. The social bond between dyads was derived in the same way using a combined data set, which contained all affiliative behaviors collected during scans together with all cases of these behaviors recorded *ad-libitum*. Only dyads with scores higher than the top quartile were considered to have a strong social bond. Rank order was calculated using submissive and dominance displays [33]. A hierarchical rank order analysis was run using Matman 1.0 software, and individual dominance ranks were estimated using the I&IS method [34] (see text S1 and Table S1 in File S1).

A second set of analysis examined the effect of several factors on the frequency and latency of elicited yawns. In the first analysis, the dependent variable was the number of yawns performed by the subjects controlled by the number of opportunities they had to observe the stimuli (entered as an offset factor). A GLMM with a Poisson error structure and a logit link function was conducted including head orientation, dominance relationship, spatial proximity, social bond, the trigger's and subject's sex, and their combination as explanatory variables. In the second analysis, the dependent variable was the time elapsed from the moment the initial yawner yawned to the moment the subject yawned. As the response variable was not normally distributed, it was transformed using a log function. A linear mixed model (LMM) was conducted entering head orientation, dominance relationship, spatial proximity, social bond, the trigger's and subject's sex, and their combination as explanatory variables. In all analyses, subject and trigger identities were included as random factors to control for repeated measures. We found no strong collinearity among the predictor variables (Pearson's and Kendall's tau r < 0.7; variance inflation factor less than three in all cases). All possible models were selected using the Akaike's Corrected Information Criterion (AICc), which identifies the most parsimonious model to explain the variance of the dependent variable. We compared the best model with the respective null model, which only contained random effects, and considered only significant effect of the individual predictors if the best model explained the variance significantly better than the null model. All analyses were performed on R version 2.14.1 [35].

## Results

### Occurrence of contagious yawning

**Figure 1:**
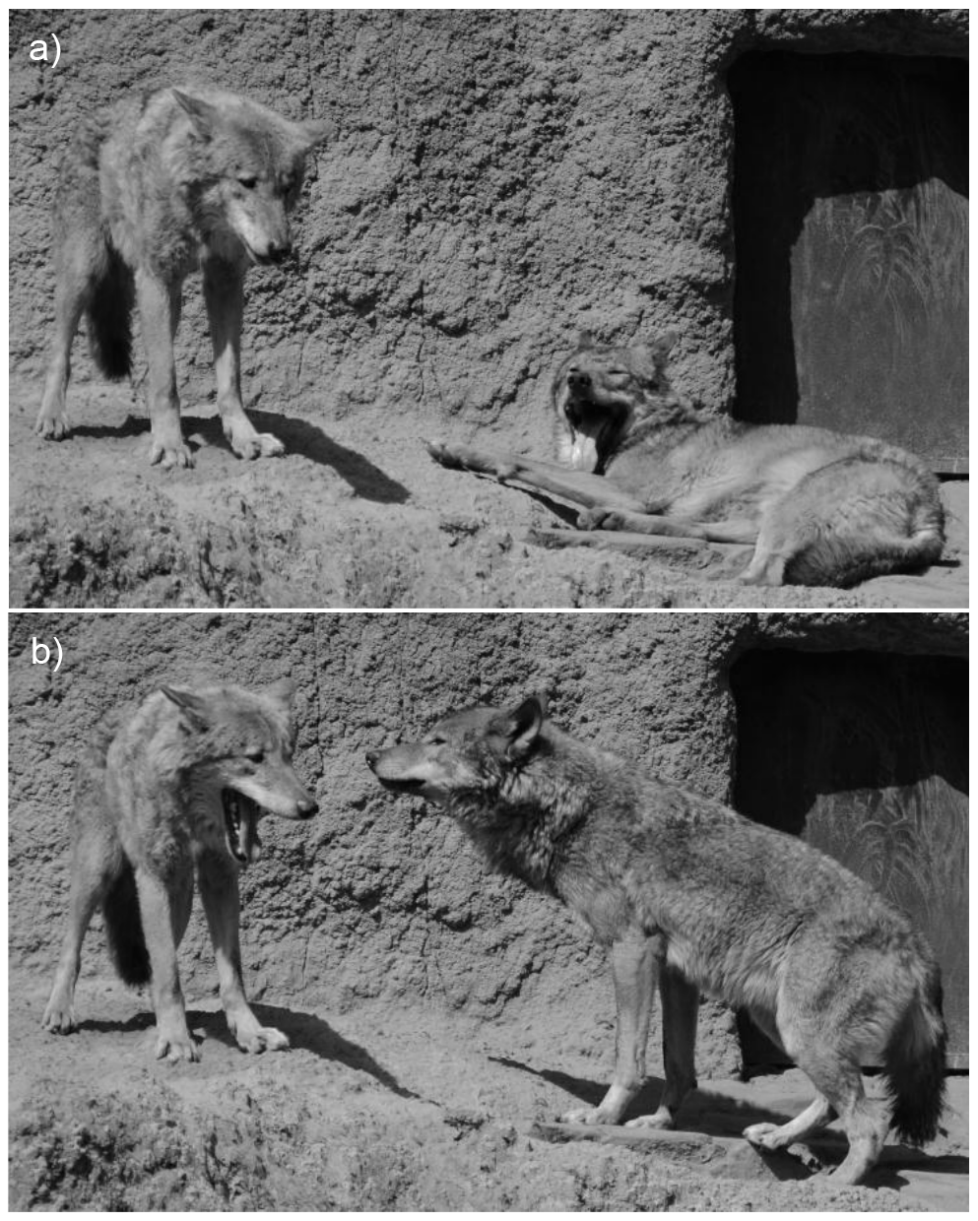
Example of contagious yawning in wolves. (a) An individual (on the right) yawned during a resting period. (b) Few seconds later, the subject (on the left) yawned contagiously. Photograph by Teresa Romero.

Subjects yawned significantly more often during the yawn condition than during the control observation (Wilcoxon signed-rank test: Z  =  −3.059, n  =  12, p  =  0.002, Figure 1, Table S1 in File S1). Furthermore, when analyzing the effect of different factors on the occurrence of contagious yawning, the best-fitting model, which fits significantly better to the data than the null model, included only two uncorrelated variables: the type of observation and the head orientation of the subject (AICc  =  1068.4, χ^2^  =  204.82, df  =  3, p < 0.001). Yawning occurred significantly more often when the subjects were exposed to the yawn stimuli, compared to when the original yawner did not perform a yawn (yawn *vs*. control observation: β  =  2.218, SE  =  0.174, z  =  12.735, p < 0.001). Additionally, yawn occurrence was also affected by the head orientation of the subjects: subjects yawned contagiously more often when they were in visual contact with the yawner and less often when the cue was auditory but not visual (facing *vs*. not within sight: β  =  −1.301, SE  =  0.291, z  =  −4.461, p < 0.001; within sight vs. not within sight: β  =  −0.908, SE  =  0.233, z  =  −3.884, p < 0.001).

### Factors affecting the frequency of contagious yawning

**Figure 2:**
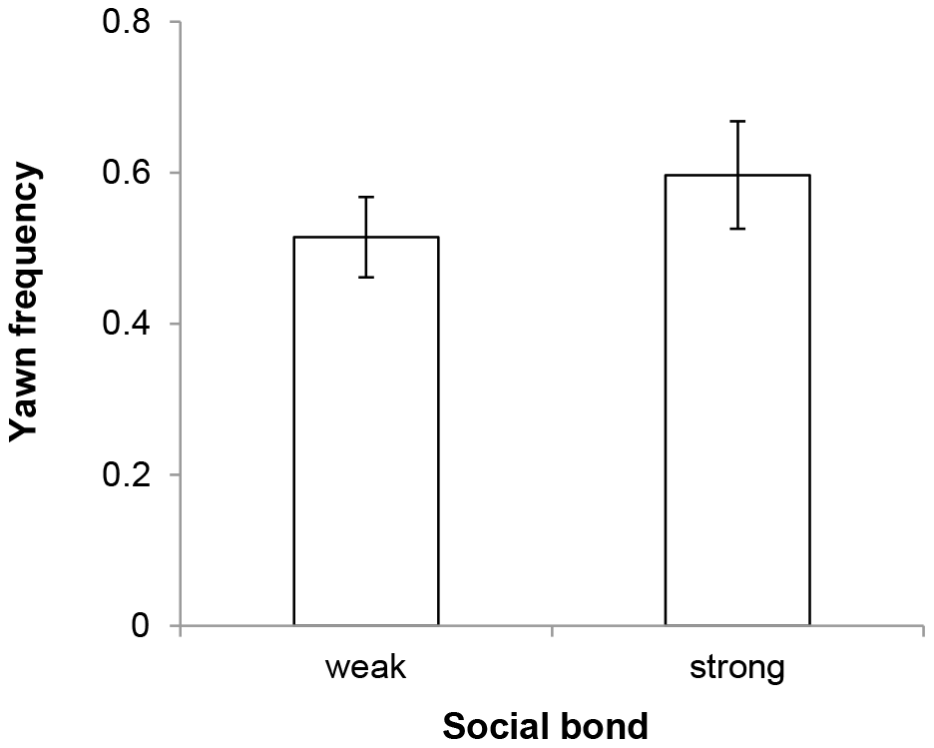
Average frequency of contagious yawning as a function of the social bond between the trigger and the subject.

When analyzing the factors that could explain the variation in the relative frequency of elicited yawns, the best-fitting model included only two predictor variables: social bond and head orientation (AICc  =  760.3, χ^2^  =  28.68, df  =  3, p < 0.001). Wolves yawned more often in response to yawns performed by close social partners than in response to yawns performed by other group members (β  =  0.375, SE  =  0.108, z  =  3.468, p < 0.001, Figure 2). Also, wolves' susceptibility to yawning was affected by their orientation towards the trigger, with subjects in visual contact yawning more frequently than those which could not see the trigger (facing *vs*. not within sight: β  =  −0.616, SE  =  0.183, z  =  −3.368, p < 0.001; within sight *vs*. not within sight: β  =  −0.573, SE  =  0.150, z  =  −3.812, p < 0.001).

### Social bond and contagious yawning

Since it is possible that receivers were only aware of the yawners' identity when they were in visual contact with them, we repeated the previous analysis including only bouts where contagious yawning was present, and the subject and the trigger were in visual contact (N  =  242). Again, wolves' susceptibility to yawn contagiously was affected by the strength of the social bond with the initial yawner (best-fitting GLMM: AICc  =  172.1, χ^2^  =  6.76, df  =  1, p  =  0.009), with wolves yawning more frequently to close social partners' yawns than to other individuals' yawns (β  =  0.277, SE  =  0.099, z  =  2.799, p  =  0.005).

**Figure 3:**
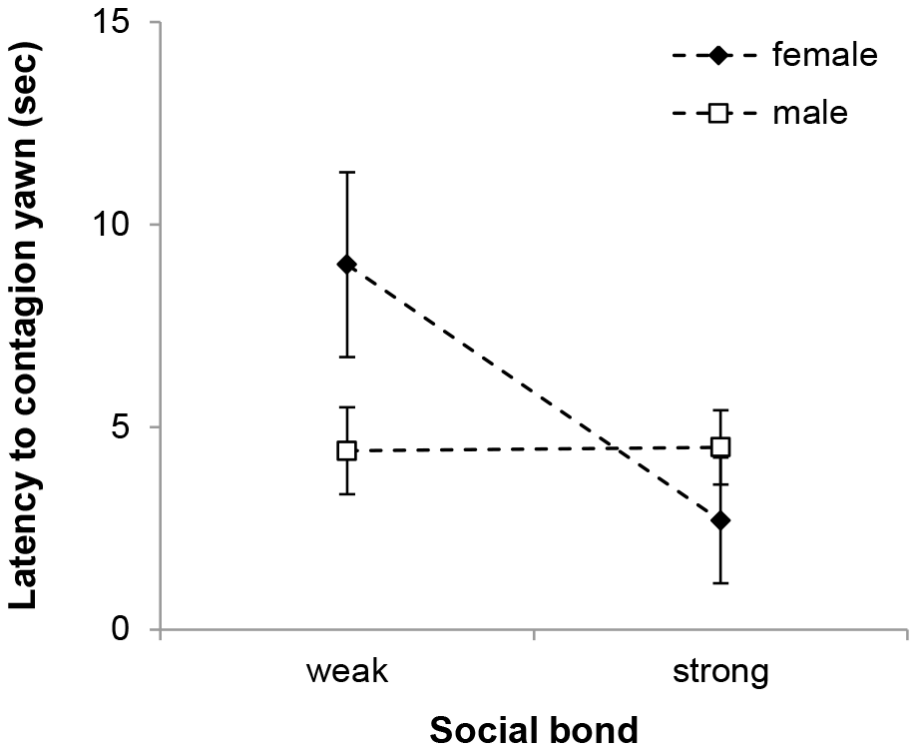
Average latency to yawn contagion for the interaction of social bond and sex of the subject.

We further investigated the effect of social bond on contagious yawning by exploring its effect on the latency to the first yawn response. Average latency to yawn contagiously was 9.0 seconds (SD  =  4.3). The LMM analysis showed that wolves' reaction time to yawn contagiously was affected by the strength of the social bond with the model. Overall, subjects' response latency increased as social bond closeness decreased (β  =  −0.217, SE  =  0.094, t  =  −2.309, p  =  0.034). This difference held only for females, however, because males' yawn latencies were not affected by the strength of the social bond with the trigger (social bond*subject's sex: β  =  0.116, SE  =  0.043, t  =  2.699, p  =  0.013, Figure 3).

## Discussion

The current study demonstrates that yawning in wolves is contagious and that, according to the empathy-based hypothesis, the strength of the social bond between the model and the subject correlated with the susceptibility to yawn contagiously. Although yawning is a widespread phenomenon among vertebrates, contagious yawning has only been documented in a few species. The communication hypothesis of contagious yawning states that yawn replication aids social animals in synchronizing behavioral and physiological states of the group [36]. For a highly social animal such as the wolf, coordinating activities has obvious adaptive advantages, since it promotes social cohesiveness of the pack. Unfortunately, we cannot directly test the communication hypothesis since yawns from motor-transitional contexts were excluded from the dataset. Studies from other taxa directly testing this hypothesis are also lacking, although indirect evidence has been found for gelada baboons [19] and domestic dogs [26]. There is also evidence that spontaneous yawning in humans and chimpanzees is related to a change in general activity levels [37], [38]. However, it remains to be seen whether yawns have any effect on the activity levels of other group members. Further research, especially in wild populations, should examine the regulating effect of yawning on synchronized group behavior in order to test its communicative function.

The present study is the first to demonstrate intraspecific contagious yawning in a carnivore species, suggesting that such ability might be deeply rooted in the Mammalia class. Although domestic dogs seem to yawn contagiously in response to human yawners [22], [25]–[27], no study has been able to demonstrate intraspecific (dog-to-dog) contagious yawning. Our finding of yawn contagion in wolves supports the notion that this ability is an adaptation for within-species social communication, which was later transferred to dog-human interactions. Furthermore, that phylogenetically distant species within the Mammalia class, i.e., primates and carnivores, are able to respond to conspecifics' yawns suggests that this response is a common ancestral trait shared by other mammalian social taxa.

Yawning has different communicative modalities (i.e., visual and audio), and although it is known that in some species yawning can be elicited via both cues [8], [10], [19], [27], [39] the exact prevalence of each modality is not clear. We found that yawn contagion occurred more frequently when the subjects were in visual contact with the initial yawner than when the trigger's yawns were out of sight. This result seems to emphasize the greater importance of visual than other sensory cues in wolves. An alternative explanation, however, is that individuals out of sight from the initial yawner were not exposed to any yawn-related stimulus. Although a yawn vocalization was sometimes audible to human observers, due to environmental constrains we were only able to reliably code visual cues as factors in affecting yawn contagion. However, the fact that significantly more yawns occurred even when the yawner was completely out of sight from the subject suggests that auditory cues might have been present and affected subjects' responses. This last result seems to indicate that contagious yawning in wolves may be elicited via auditory cues, which is in line with the idea that motor facilitation in human and non-human animals can be activated by a variety of sensory modes [36], [40], [41]. For instance, even reading about or thinking about yawns trigger yawns in humans [36].

The present study supports an empathy-based explanation of contagious yawning in wolves, as yawns occurred disproportionately when the stimulus was produced by parties socially close to the observer. These differences hold after statistically controlling for time spent in close spatial proximity and attention to the initial yawner. These last results indicate that contagious yawning in wolves is not mediated by the mere opportunity of observing the yawns of others, but rather underscored by affective components of the behavior. In both human and other animals, empathy is not equally aroused by the emotional signals of any individual, but rather is facilitated by similarity, familiarity, and social closeness [1], [4], [42], [43].

According to the Perception-Action model [4], the observation of another's emotional states automatically and unconsciously activates neural representations of similar states in the observer. The more similar and socially close two individuals are, the easier the identification with the partner [1]. In line with this hypothesis, several brain regions linked to contagious yawning are implicated in the simulation of actions, social behavior, and empathy [10], [11]. That the social closeness predicts the infectiousness of yawning in wolves is consistent with the hypothesis that this phenomenon is mediated by empathy. Thus, our results trace back to carnivores the link between contagious yawning and empathy, supporting the idea that basic building blocks of empathy might be present in a wide range of species.

Another point in favor of the empathy hypothesis is the observed sex differences in reaction time. Overall, female wolves responded quicker than males when the initial yawner was a close associate, suggesting that females were particularly responsive. Furthermore, the sex pattern observed in this study did not reflect simply sex differences in sociability, because in wolf society in general, as well as in our study in particular, females are not more affiliative than males (mean ±SD percentage of scan samples individuals affiliated with any group members: females, 6.72±3.31%; males, 7.23±3.26%; Mann-Whitney U-test, N_f_  =  6, N_m_  =  6, U  =  17, z  =  −0.0801, p  =  0.532). Although sexual dimorphism of yawning frequency has not been observed in humans [36], [44], our finding paralleled results from gelada baboons, where females, but not males, tend to match the type of yawn they observed [19]. Although our results should be taken with caution due to our small sample size, the observed sex difference in reaction time probably reflects the higher ability of female wolves to react to the emotional stimulus of their close associates.

In conclusion, this study provides the first evidence of intra-specific contagious yawning in a carnivore species, the wolf, which suggests that such ability may be widespread among mammals. In addition, our findings show that this phenomenon is modulated by the degree of bonding between individuals. In humans, conscious or unconscious matching of behaviors and facial expressions of others has been theorized to be central in emotionally connecting two individuals [45], [46]. Recently, this idea has received support from behavioral studies in monkeys [47], [48]. Therefore, to yawn when a social partner yawns could be advantageous to promote social connections and affiliative behaviors among individuals.

While an observational study cannot determine the exact cause-effect relationship, our results indicate that contagious yawning is modulated by emotional components of the behavior. These results paralleled previous observations in primates and domestic dogs, and are consistent with the claim that the mechanism underlying contagious yawning relates to the capacity for empathy, an ability that humans probably share with other species beyond primates. By demonstrating the occurrence of contagious yawning in a phylogenetically distant taxon and providing insights into the mechanism underlying this phenomenon, this study broadens our understanding of the evolutionary history of empathy.

## Supporting Information

File S1 **Supporting methods (Text S1) and tables (Tables S1 and S2).**
(DOCX)
