# Supplementary material for: Social Modulation of Contagious Yawning in Wolves
Source: PLoS One. 2014 Aug 27;9(8):e105963. doi: 10.1371/journal.pone.0105963 (PMC4146576; doi:10.1371/journal.pone.0105963)
Supplement: File S1 — Supporting methods (Text S1) and tables (Tables S1 and S2). (DOCX) [file pone.0105963.s001.docx]

**SUPPORTING INFORMATION**

**Social modulation of contagious yawning in wolves**

Teresa Romero^1,2,^*, Marie Ito^1^, Atsuko Saito^1^, Toshikazu Hasegawa^1^

^1^Department of Cognitive and Behavioral Sciences, The University of Tokyo, Komaba 3-8-1, Meguro, Tokyo, Japan

^2^Japan Society for the Promotion of Science, Kojimachi 5-3-1, Chiyoda, Tokyo, Japan

*Corresponding author’s address: T. Romero, Department of Cognitive and Behavioral Sciences, Graduate School of Arts and Science, The University of Tokyo, Komaba 3-8-1, Meguro, Tokyo 153-8902, Japan. email: [tromero@darwin.c.u-tokyo.ac.jp](mailto:tromero@darwin.c.u-tokyo.ac.jp)**Supporting Methods**

**Text S1. Hierarchical Rank Order**

We evaluated two combined measures of behaviours expressing dominant or submissive relationships (table S2) [1] in carrying out our hierarchical rank order analysis. For each combined measures, the linearity of the hierarchy was examined using the improved version of Landau’s index of linearity (h’) [2], which corrects for unknown and tied relationships. This index ranged from 0 (non-linear) to 1 (highly linear). To determine the statistical significance of h’, a two-step randomization process with 10000 randomizations was performed using MatMan [2]. When significant linearity was found, dominance ranks were determined using the I&SI method [3]. The directional consistency index (DC), i.e. the directional frequency of the behaviour in respect to the total occurrence of it, was also calculated using the formula: DC = (H - L) / (H + L), where H is the number of times a behaviour occurred in the most frequent direction within each dyad, and L is the number of times occurring in the less frequent direction. This directional consistency index ranges between 0 (completely bidirectional) to 1 (completely unidirectional). A behaviour would qualify as a dominance signal if it is expressed in most dyads in a predominantly unidirectional manner, and is expressed in most of the relationships in the group [2].

The combined measure of dominant behaviours showed a high directional consistency (DC = 0.78) and a significant but low linearity index (h’ = 0.46, χ2 = 32.87, d.f. = 20.62, p = 0.032). Furthermore, its coverage was not good with 20 out of 66 blank relationships. On the other hand, the combined measure of submissive behaviours was a good dominance measure since it had a high and significant linearity index (h’ = 0.84, χ2 = 63.37, d.f. = 20.62, p < 0.001), showed a high directional consistency (DC = 0.95) and in only 9% of the dyads the relationship was unknown. Thus, we used this measure to derive the rank order of the individuals (table S1).

**Supporting References**

1 Mech, L. D. & Boitani, L. *Wolves: Behavior, Ecology, and Conservation*. Chicago, IL: University of Chicago Press (2003).

2 De Vries, H. A. N. An improved test of linearity in dominance hierarchies containing unknown or tied relationships *Anim. Behav.* **50**, 1375-1389. (1995)

3 De Vries, H. A. N. Finding a dominance order most consistent with a linear hierarchy: a new procedure and review *Anim. Behav.* **55**, 827-843. (1998)

**Supporting Tables**

**Table S1**. Age, sex, rank order, and yawning rate (i.e. proportion of observations in which yawning occurred)

| **Subject** | **Sex** | **Age** | **Rank** | **Yawning** | **rate** |
| --- | --- | --- | --- | --- | --- |
| **code** |  | **(months)** | **order** | **yawning observation** | **control observation** |
| LB | Male | 149 | 1 | 0.31 | 0.09 |
| MO | Female | 173 | 2 | 0.47 | 0.03 |
| MI | Female | 91 | 10 | 0.62 | 0.25 |
| CH | Female | 78 | 9 | 0.58 | 0.15 |
| CL | Male | 65 | 7 | 0.73 | 0.16 |
| RT | Male | 65 | 6 | 0.42 | 0.10 |
| RY | Male | 65 | 4 | 0.41 | 0.15 |
| MA | Female | 65 | 12 | 0.36 | 0.06 |
| NE | Male | 54 | 3 | 0.44 | 0.09 |
| LK | Male | 54 | 5 | 0.33 | 0.19 |
| LI | Female | 54 | 8 | 0.54 | 0.06 |
| ME | Female | 54 | 11 | 0.64 | 0.05 |

**Table S2**. Dominance-related behaviours. Submissive behaviours were given by subordinate (S) towards dominant individuals (D), while dominant behaviours were directed from dominants to subordinate individuals

| **Subordinate behaviours** |  |
| --- | --- |
| active submission | subject S approaches subject D with wagging tail, looks up and whimpers with lowered posture |
| passive submission | subject S rolls over to show stomach, tail wagging, at approach of subject D |
| mouth lick | subject S repeatedly licks mouth area of D |
| **Dominant behaviours** |  |
| stand over | subject D sits or lays over with at least 20% of subject’s torso over subject S’ body |
| mouth grasp | subject D growls and grasps subject S's muzzle by mouth |
| neck grasp | subject D growls and grasps subject S's neck by mouth |
